# Different operators and histologic techniques in the assessment of germinal center-like structures in primary Sjögren’s syndrome minor salivary glands

**DOI:** 10.1371/journal.pone.0211142

**Published:** 2019-01-25

**Authors:** Francesco Carubbi, Alessia Alunno, Paola Cipriani, Gino Coletti, Barbara Bigerna, Mirko Manetti, Paola Di Benedetto, Onelia Bistoni, Gianluca Cipolloni, Vasiliki Liakouli, Piero Ruscitti, Elena Bartoloni, Roberto Giacomelli, Roberto Gerli

**Affiliations:** 1 Rheumatology Unit, Department of Biotechnological and Applied Clinical Science, School of Medicine, University of L'Aquila, Italy; 2 Department of Medicine, ASL1 Avezzano-Sulmona-L’Aquila, L’Aquila, Italy; 3 Rheumatology Unit, Department of Medicine, University of Perugia, Perugia, Italy; 4 Pathology Unit, Biomedical Department, ASL1 Avezzano-Sulmona-L’Aquila, L'Aquila, Italy; 5 Institute of Haematology, Department of Medicine, University of Perugia, Perugia, Italy; 6 Department of Experimental and Clinical Medicine, Section of Anatomy and Histology, University of Florence, Florence, Italy; University of Bergen, NORWAY

## Abstract

**Objective:**

A standardization of minor salivary gland (MSG) histopathology in primary Sjögren’s syndrome (pSS) has been recently proposed. Although there is strong agreement that germinal center (GC)-like structures should be routinely identified, due to their prognostic value, a consensus regarding the best protocol is still lacking. Aim of this study was to compare the performance of different histological techniques and operators to identify GC-like structures in pSS MSGs. MSG biopsies from 50 pSS patients were studied.

**Methods:**

Three blinded operators (one pathologist and two rheumatologists with different years of experience in pSS MSG assessment) assessed 50 MSGs of which one slide was stained with haematoxylin and eosin (H&E) and consecutive slides were processed to investigate CD3/CD20, CD21 and Bcl-6 expression.

**Results:**

By assessing 225 foci, the best agreement was between H&E-stained sections evaluated by the rheumatologist with more years of experience in pSS MSG assessment and CD3/CD20 segregation. In the foci with CD21 positivity, the agreement further increased. Bcl-6^-^ foci could display a GC, detected with other staining, but not vice versa.

**Conclusion:**

GC assessment on H&E-stained sections should be performed with caution, being operator-dependent. The combination of H&E with CD3/CD20 and CD21 staining should be recommended as it is reliable, feasible, able to overcome the bias of operator experience and easily transferrable into routine practice.

## Introduction

Primary Sjögren’s syndrome (pSS) is a chronic inflammatory disease mainly affecting exocrine glands [1]. Minor salivary gland (MSG) biopsy is widely used in the classification criteria of pSS [2,3], and the histopathological hallmark of the disease is the focal lymphocytic sialadenitis (FLS), with sensitivity and specificity >80% [4]. Aggregates of at least 50 infiltrating lymphocytes (namely foci) are often organized into tertiary ectopic lymphoid structures (ELS), showing segregated T- and B-cell zones, high endothelial venules and networks of CD21^+^ follicular dendritic cells (fDCs). As recently reviewed, some of these structures display functional features of classical germinal centers (GCs) such as the expression of the enzyme activation-induced cytidine deaminase (AID) and in situ B cell affinity maturation and clonal selection [5]. The formation of ELS is not peculiar of pSS as they can be observed in other rheumatic diseases or organ specific autoimmune conditions, solid tumors, chronic infection and graft rejection [5]. In recent years, several studies explored the presence and features of GC-like structures in MSGs and parotid gland of patients with pSS. From most of these studies, it emerged that the detection of GC-like structures in the lymphoid infiltrates of the salivary glands (SG) is clinically relevant, since the presence of these structures seems to be associated with more severe disease [6–8], and higher risk of lymphoma development [9, 10]. However, previous studies aimed at assessing GC-like structures in pSS reported a highly variable prevalence ranging from 18% to 67% based on different methods [6–8]. In particular, the peculiar structure of fully formed GCs, namely a well-distinguished light and dark zone segregation, detectable with haematoxylin and eosin (H&E) staining, is sufficient to allow pathologists to detect these structures in secondary lymphoid organs. However, when GCs ectopically develop in non-lymphoid tissues such as MSGs, this detection is more challenging since the classical dark/light zone may lack and only a lighter are within the follicle can be present. Therefore, additional GC-specific immunostainings, such as B-cell lymphoma (Bcl)-6, CD21 and AID, have been suggested to assess ELS in MSGs [11]. Recently, the EULAR Sjögren’s syndrome study group (eSSential) provided standardized consensus guidance for the use of MSG histopathology in classification and clinical trials [12]. Although there is strong agreement that the presence of GC-like structures should be reported in routine clinical practice, the consensus regarding which technique should be employed is lacking [11]. The lack of consensus, in association with the difficulty to identify these structures and the variability of pSS samples used in the literature (i.e. parotid glands versus MSG samples), may explain the different prevalence of GC-like structures in pSS biopsies [6], and different associations found from both a pathological and clinical point of views [7, 9, 10, 13, 14]. The harmonization of GC-like structure assessment and, subsequently, the development of recommendations to be used in clinical practice are compelling as proven by the ongoing intense debate in the literature [11, 15, 16]. On this basis, the purpose of our study was to compare, for the first time, the performance of different histological techniques and operators with variable histopathology expertise in the assessment of GC-like structures in MSGs of pSS patients.

## Materials and methods

### Study population and sample assessment

Fifty patients with pSS fulfilling the 2002 American-European classification criteria, including the histological criterion, were enrolled [2]. Four formalin-fixed, paraffin-embedded consecutive sections measuring 3 μm in thickness from each MSG biopsy collected at the time of the diagnosis were used for this study. One slide was dewaxed, rehydrated and stained with H&E. Three blinded operators (Op1#, AA, rheumatologist with <3 years of experience in pSS MSG assessment; Op2#, FC, rheumatologist with >5 years of experience in pSS MSG assessment; Op#3, GCo, pathologist with 35 years of experience in general SG pathology) scored these H&E stained slides for the presence or absence of GC-like structures (yes/no) in each focus. Consecutive slides of each biopsy were processed for immunofluorescence to identify B/T-cell segregation by CD3/CD20, the fDC network by CD21 as described elsewhere [7]. To assess Bcl-6, sections were sent to the laboratory of Perugia University where the anti-Bcl-6 Ab clone, used for this study, was developed (clone PG-B6p, available from Dako, Denmark) [17]. Sections were then processed with PT link for deparaffinization, rehydratation and heat-based antigen retrieval using the Target Retrieval Solution pH 9 (Dako, Denmark). Subsequently, sections were processed using an autostainer with the Dako REAL^TM^ Detection System, Alkaline Phosphatase/RED, mouse (Dako, Denmark). All foci detected in each MSG section were separately evaluated and scored. GC-like structures in H&E stained sections were defined as lighter areas within the lymphoid infiltrate composed of both lymphoid cells (centrocytes, centroblasts) and cells of non-lymphoid nature (macrophages and fDCs) [15]. A focus displaying a clear partition of a B cell area and a T cell area was considered positive for B/T cell segregation [7]. The presence of a CD21^+^ network or a cluster of ≥5 adjacent Bcl6+ within a focus, were classified as GC-like structures based on previously published papers [7, 13]. Tonsil specimens were used as positive control for the staining protocols.

### Ethical approval

The local ethics committee (CEAS Umbria) approved this study. All patients provided written informed consent in accordance with the Declaration of Helsinki.

### Statistical analysis

Data were analysed with SPSS 23 software. Different tests were performed to assess the agreement/disagreement of: i) the operators vs each other on H&E scoring; ii) the operators vs the objective histological techniques; iii) the objective histological techniques vs each other. Cohen’s kappa was calculated to identify the agreement, McNemar chi square test was calculated to evaluate the asymmetry of disagreement.

## Results

A total of 225 foci from 50 MSGs were scored. The focus score (FS) ranged from 1 to 12 (namely confluent foci) with a mean value of 2.02±2.2 (standard deviation) and a median value of 1.45. The number of positive MSG samples, according to the presence of at least one focus scored as possible GC-like structure by the operators on H&E or by different histological techniques (alone or in combination), is shown in Table 1.

**Table 1:** Number of positive minor salivary glands (MSG) samples according to the presence of at least one focus scored as possible germinal center (GC)-like structure by the operators (Op) on haematoxylin and eosin (H&E) or by different histological techniques.

Operators and histological techniques	Number (%) of positive MSG samples
Op#1 H&E	24/50 (48)
Op#2 H&E	29/50 (58)
Op#3 H&E	13/50 (26)
CD3/CD20 segregation	25/50 (50)
CD21^+^	26/50 (52)
Bcl-6^+^	13/50 (26)
CD3/CD20 segregation AND CD21^+^	20/50 (40)
CD3/CD20 segregation AND Bcl-6^+^	2/50 (4)
CD21^+^ AND Bcl-6^+^	0/50 (0)
CD3/CD20 segregation AND CD21^+^ AND Bcl-6^+^	11/50 (22)

**Fig 1:**
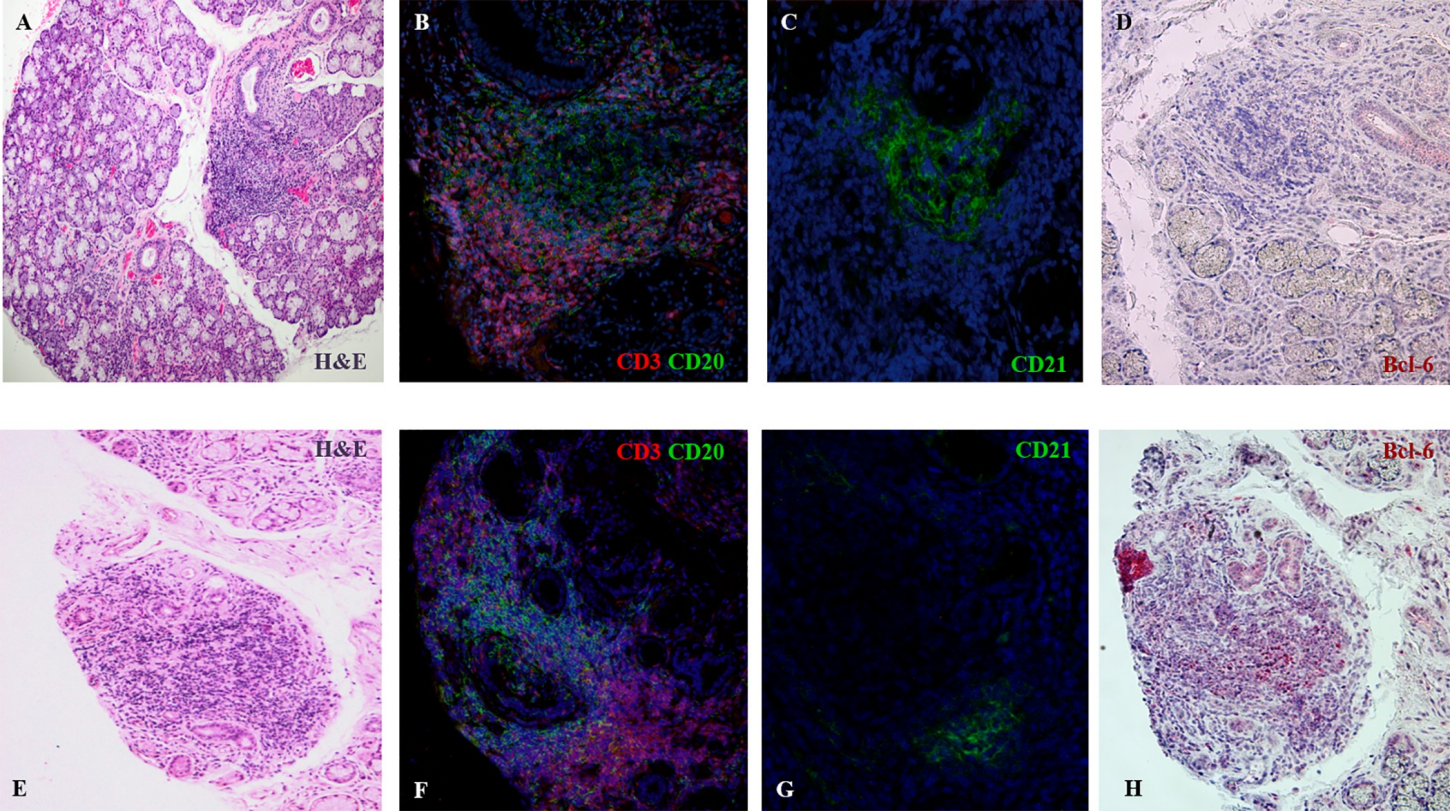
Representative microphotographs of 2 pSS MSGs stained with haematoxylin and eosin (A, E), CD3/CD20 (B, F), CD21 (C, G) and B-cell lymphoma (Bcl)-6 (D, H). In panel A, a light zone surrounded by a dark zone is detected (square) which allows to identify a possible GC-like structure. Panels B and C show the presence of B/T cell segregation and a follicular dendritic cell (fDC) network. However, Bcl-6 is absent (D). In panel E, there are no hallmarks of GC-like structures, however there is B/T cell segregation (F), a fDC network (G) and Bcl-6 positivity (H).

**Fig 2:**
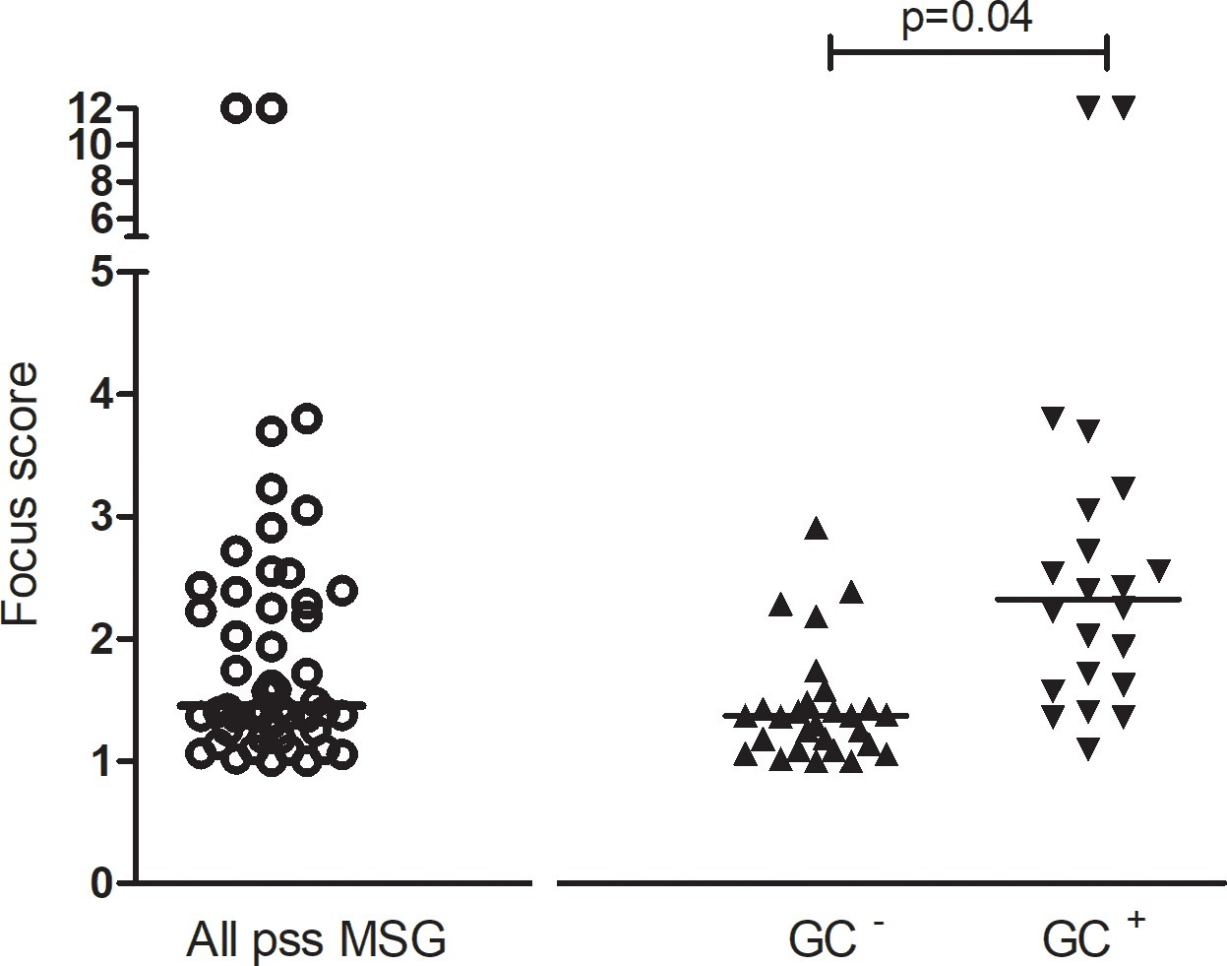
Distribution of focus score values in all MSG samples and according to the presence or absence of germinal center-like structures.

We found a wide variability in the identification of GC-like structures that is both operator and technique-dependent. The assessment of H&E slides by operators with different years of experience accounts for different identification of GC-like structures. However, the presence of CD3/CD20 segregation and CD21 positivity seems to identify the highest prevalence of GC-like structures (Fig 1). Fig 2 displays the overall focus score values and their distribution in MSGs with or without GC-like structures (as defined by the presence of at least one focus with CD3/CD20 segregation and CD21 positivity). In line with previous studies, we confirmed that higher focus score values are associated with the presence of GC-like structures (7, 14).

**Fig 3:**
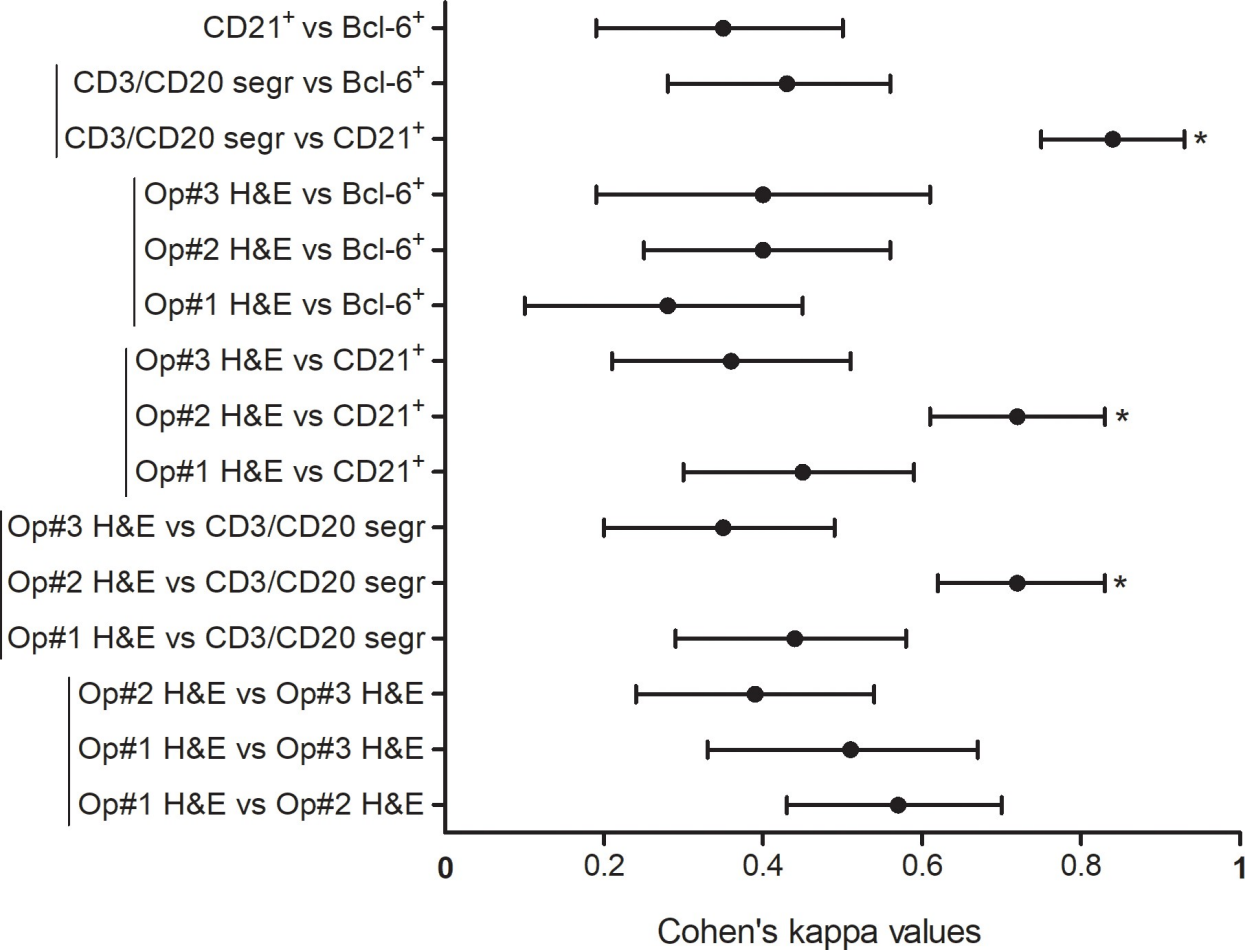
Forest plot displaying the Cohen’s kappa values and respective 95% confidence intervals among operators and histological techniques. Vertical lines identify subgroups and asterisks display statistically significant differences within the subgroups (p<0.05). (Op: operator; H&E: haematoxilin and eosin; segr: segregation).

The agreement between the 3 operators on CD3/CD20, CD21 and Bcl-6 stainings was perfect (all Cohen’s kappa values = 1), confirming that these are objective methods that do not generate uncertainty whether samples are positive or negative. Conversely, the agreement between the 3 operators on H&E stained sections ranged from fair (0.39) to moderate (0.57), without any significant difference. Furthermore, the agreement between the scores on H&E stained sections by Op#2, and the presence/absence of B/T cell segregation was significantly higher when compared to Op#1 and Op#3 (0.72, 0.44 and 0.35 respectively, p<0.05). A similar behaviour was also observed regarding the agreement between Op#2 and the presence/absence of the CD21^+^ staining, when compared to Op#1 and Op#3 (0.72, 0.45, 0.36 respectively, p<0.05). Among the 3 methods employed, the best agreement was observed between B/T-cell segregation and the positivity for CD21 staining (0.84). Combining the results obtained by B/T-cell segregation and CD21^+^ staining (namely assigning a positive value to foci displaying both B/T-cell segregation and CD21^+^ staining) and calculating the Cohen’s kappa with the operators, the agreement slightly increased (Op#1 = 0.47, Op#2 = 0.75, and Op#3 = 0.40) (Fig 3).

S1 Fig shows the scoring of each focus by the 3 operators on H&E stained sections and the 3 objective techniques.

Subsequently, we aimed at evaluating whether the disagreement between operators and operators versus methods was significant and symmetric, or whether there was a difference between positive and negative samples. Table 2 shows the p values of the Mc Nemar test.

**Table 2:** p values resulting from the Mc Nemar test to identify the distribution of disagreement among operators (Op) and histological techniques. Significant p values <0.05 identify asymmetric disagreement. H&E, haematoxylin and eosin.

Operators and histological techniques	Mc Nemar test p value
Op#1 H&E vs Op#2 H&E	0,008
Op#1 H&E vs Op#3 H&E	<0.0001
Op#2 H&E vs Op#3 H&E	<0.0001
Op#1 H&E vs CD3/CD20 segregation	0.014
Op#2 H&E vs CD3/CD20 segregation	1
Op#3 H&E vs CD3/CD20 segregation	<0.0001
Op#1 H&E vs CD21^+^	0.029
Op#2 H&E vs CD21^+^	1
Op#3 H&E vs CD21^+^	<0.0001
Op#1 H&E vs Bcl-6^+^	0.09
Op#2 H&E vs Bcl-6^+^	<0.0001
Op#3 H&E vs Bcl-6^+^	0.115
CD3/CD20 segregation vs CD21^+^	0.774
CD3/CD20 segregation vs Bcl-6^+^	<0.0001
CD21^+^ vs Bcl-6	<0.0001

As far as the comparison between operators on H&E stained sections is concerned, the disagreement was always asymmetric. In detail, a high number of samples classified as positive by Op#2 were classified as negative by Op#1, while a consistent number of samples classified as negative by Op#3 were classified as positive by Op#1 and Op#2. With regard to the comparison between operators on H&E vs other histological techniques, Op#1 and Op#3 classified as negative a significant number of samples where B/T-cell segregation was observed. On the other hand, the disagreement between Op#2 and B/T-cell segregation or CD21^+^ staining was negligible and symmetric between negative and positive samples. To note, the 2 methods with the highest agreement, B/T-cell segregation and CD21^+^ staining, did not disagree significantly and there was no significant difference between positive and negative samples.

## Discussion

To our knowledge, this is the first study assessing the agreement and disagreement as well as the contribution of operators’ experience for the proposed procedures to identify GC-like structures, in a cohort of pSS patients. In fact, standardization of histopathological patterns in pSS MSGs, including the presence of GC-like structures, is still a major unmet need as confirmed by the high variability of GC-like structures prevalence, detected by each of the currently available techniques [11, 15, 16]. Overall, the prevalence of GC-like structures in MSG specimens (namely the presence of at least one focus positive for at least one operator or histological technique) ranged between 26 and 52% according to the different operator and to the fact that different staining protocols identify different cell populations. When assessing each focus separately, the best agreement was observed between Op#2 and B/T cell segregation. In this regard, our study shows that the H&E scoring by a rheumatologist with over 5 years of experience in pSS MSG histopathology, ensured the identification of almost 80% of foci with B/T-cell segregation. When considering the co-existence of B/T cell segregation and CD21 positivity in the same focus, this agreement reached 86%. CD21 is expressed by fDCs, but the presence of these cells does not necessarily imply that GCs are present. In fact, lymphoid infiltrates in SG tissue of patients with pSS may contain fDC networks in the absence of GCs [18], with consequent overestimation of the number of GCs in SG tissue using only CD21 staining. On the basis of these observations, and according to Fisher et al. who recommended to perform H&E, CD3/CD20 and CD21 staining, our paper reinforces that H&E staining should not be performed alone, in order to prevent operator-dependent bias [12]. Furthermore, the presence of fDC network together with B/T-cell segregation would be expected in all GC-like structures, and this combined approach would avoid the risk of overestimating GC-like structures if relying on CD21 alone [12]. H&E is considered sufficient to allow accurate detection of a fully formed GC by experienced pathologists, in particular in normal secondary lymphoid organs or lymphomatous tissues, with a well-distinguished light and dark zone segregation. Conversely, in MSGs this detection is more challenging and GCs are often only appreciable as a lighter area within the follicular structure, without the classical dark/light zone segregation. In our study, we defined GCs in H&E-stained sections as lighter areas within the lymphoid infiltrate composed of both lymphoid cells (centrocytes, centroblasts) and cells of non-lymphoid nature (macrophages and fDCs), as previously described [15]. Interestingly, we noticed that the pathologist tended to underestimate the number of possible GC-like structures compared to other operators, probably due to the lack of classical dark/light zone segregation. Conversely, the rheumatologist with over 5 years of experience may tend to overestimate. This may also explain the fact that in our study the pathologist is the operator with the highest agreement with Bcl-6 positivity. Bcl-6 is a transcription factor consistently expressed at high levels by GC B-cells. Although it is routinely used worldwide in pathology laboratories for the diagnosis of lymphoma, the applicability of staining protocols for such nuclear factor has not been extensively validated in other tissues [12]. In our study, the agreement between Bcl-6^+^ staining, B/T-cell segregation and CD21 positivity was poor. Bcl-6 was positive in less than 40% of foci showing B/T cell segregation and CD21 positivity. To note, however, the foci showing this triple positivity were more frequently scored as GC-like structures by the pathologist compared to those where B/T-cells were segregated, CD21 staining was positive, but Bcl-6 was not detectable. Furthermore, isolated Bcl-6^+^ staining was detected in the 2% of foci where B- and T-cells were not segregated and CD21 staining was negative. Interestingly, these foci were scored as no GC-like structures by the three operators. Although Delli et al proposed Bcl-6 as an unequivocal marker of GC [15], our data pointed out that the absence of Bcl-6 does not necessarily rule out the presence of the GC. These observations, together with the well-recognized technical difficulties that may occur with nuclear staining protocol for Bcl-6, make this marker less likely to be considered for routine assessment of GC-like structures in MSGs in clinical practice. It should be noted, however, that our study is focused only on MSGs, being the sample routinely collected in the majority of Rheumatology centres. Hence, an additional value of Bcl-6 staining in parotid gland cannot be excluded. Finally, it is of interest the finding that activation-induced cytidine deaminase (AID) seems to be expressed if the GCs are functional [9]. However, accurate staining for this marker is technically challenging and, therefore, its use appears to be confined, at the moment, only to research fields rather than routine clinical practice [9]. Another aspect deserving consideration pertains to the possible morphological differences outlined at different cutting levels. We acknowledge that studies conducting for research purpose always include samples from different cutting levels. However, this is not always the usual procedure in real life setting for diagnostic purposes. To overcome this discrepancy, the definition of cutting levels as a standardised requirement for a satisfactory assessment of MSGs would facilitate the implementation of this aspect in daily practice.

Our study displays some limitations. In particular, the study looks at the ability of only 3 operators to assess GC-like structures, but the interpretation of these results is open to potential bias based on the selection of the individuals reviewing the cases. This study results from the shared experience of 2 Italian centers and needs validation on international level.

In conclusion, although performed on a small cohort, our study is the first to suggest that the detection of GC-like structures on H&E staining sections should be performed with caution as it is operator-dependent. Furthermore, our data support the need to combine H&E with additional immunostaining to overcome such bias. In this setting, the combined staining of CD3/CD20 to identify B/T-cell segregation and CD21 to identify the fDC network seems to represent the most reliable approach that could be easily translated into clinical practice. In this regard, a standardization of staining protocols along with a closer collaboration between rheumatologists and pathologists would be desirable. In fact, the detection of GC-like structures in the lymphoid infiltrates of the salivary glands (SG) is clinically relevant due to the potential prognostic value of such lesions. The presence of GC-like structures seems to be associated with more severe disease [6–8], and higher risk of lymphoma development [9, 10] and therefore it is ultimately advisable to explore the prognostic significance and implications of different staining protocols in large cohorts,.

## Supporting information

S1 Fig The plot displays how each focus was scored by Op#1 (A), Op#2 (B), and Op#3 (C) on H&E stained sections (red = positive; blue = negative), and the presence (red) or absence (blue) of CD3/CD20 segregation (D), CD21 (E) and Bcl-6 (F).
(TIF)
