# Supplementary figures and images for: Different operators and histologic techniques in the assessment of germinal center-like structures in primary Sjögren’s syndrome minor salivary glands
Source: PLoS One. 2019 Jan 25;14(1):e0211142. doi: 10.1371/journal.pone.0211142 (PMC6347225; doi:10.1371/journal.pone.0211142)

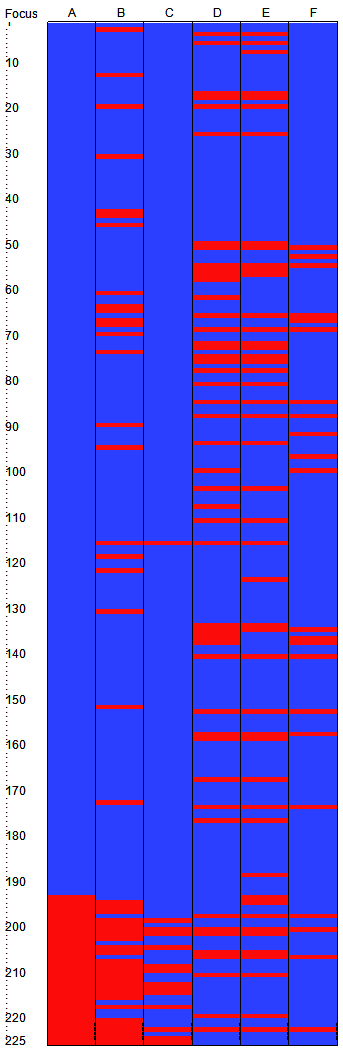

Supplement: S1 Fig — The plot displays how each focus was scored by Op#1 (A), Op#2 (B), and Op#3 (C) on H&E stained sections (red = positive; blue = negative), and the presence (red) or absence (blue) of CD3/CD20 segregation (D), CD21 (E) and Bcl-6 (F). (TIF) [file pone.0211142.s001.tif]
